# Supplementary material for: Causal Relationship between Aging and Anorexia Nervosa: A White-Matter-Microstructure-Mediated Mendelian Randomization Analysis
Source: Biomedicines. 2024 Aug 16;12(8):1874. doi: 10.3390/biomedicines12081874 (PMC11351342; doi:10.3390/biomedicines12081874)
Supplement: Supplementary file 1 [file biomedicines-12-01874-s001.zip › Supplementary Figure/Figure S8/Leaveoneout plot of MR analysis for TL to ubm-b-1733.pdf]

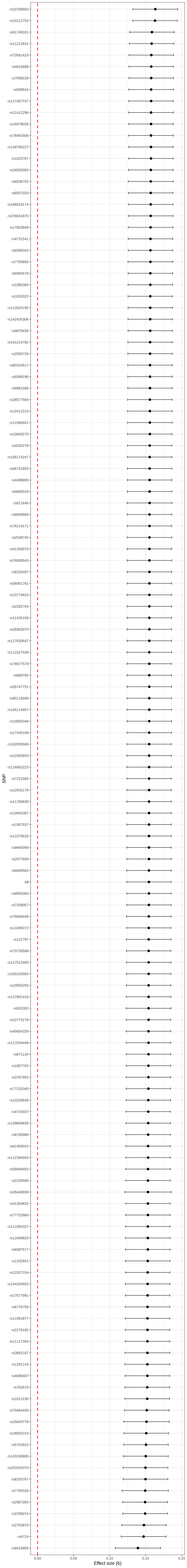

rs10768683
rs10112752
rs61748181
rs11212631
rs73581419
rs4616688
rs7099229
rs939916
rs117407747
rs11412296
rs16978028
rs78491606
rs139795227
rs1023767
rs34550383
rs6536702
rs6007020
rs188918174
rs376641875
rs17803849
rs4731541
rs6590343
rs7790856
rs6584579
rs1985369
rs1003322
rs113525195
rs142426306
rs9878436
rs141214782
rs2056726
rs80324517
rs9398196
rs6681568
rs28577594
rs12412214
rs11584821
rs10840270
rs4530278
rs185174247
rs66731853
rs4498805
rs9600019
rs611646
rs6659669
rs76219171
rs2538745
rs41269079
rs76065543
rs8102497
rs56061761
rs10774624
rs2282764
rs11426156
rs28363070
rs117630647
rs111527438
rs79977579
rs869785
rs55747751
rs80116508
rs145114957
rs10805346
rs17445108
rs182059586
rs12925933
rs116863223
rs7221585
rs12932179
rs11769630
rs10845367
rs1957937
rs11579626
rs9940099
rs2977608
rs6669563
All
rs9955360
rs7209057
rs76666449
rs11085072
rs131797
rs73730598
rs117512405
rs150150565
rs10905255
rs137901416
rs932002
rs10773176
rs45604339
rs117034449
rs871134
rs1907702
rs3767952
rs77231040
rs13230646
rs4743037
rs139669835
rs6790988
rs61405042
rs112394943
rs59409453
rs2230590
rs35446936
rs41304832
rs77732866
rs111950327
rs11699829
rs6587577
rs1332941
rs11557154
rs144204502
rs17677991
rs6776756
rs11991877
rs2276182
rs11117354
rs3891167
rs1291143
rs4695407
rs762679
rs1611236
rs75664430
rs35640778
rs28502153
rs5742915
rs143190905
rs202034370
rs8105767
rs7705526
rs2967355
rs3785074
rs2763979
rs4724
rs9419958
SNP
0.00
0.05
0.10
0.15
0.20
Effect size (b)
